# The prediction model of suicidal thoughts in Korean adults using Decision Tree Analysis: A nationwide cross-sectional study

**DOI:** 10.1371/journal.pone.0223220

**Published:** 2019-10-09

**Authors:** Sung-Man Bae

**Affiliations:** Department of Psychology and Psychotherapy, College of Health Science, Dankook University, Cheonan, Chungnam, Republic of Korea; University of Toronto, CANADA

## Abstract

The purpose of this retrospective decisional analysis study is to develop the prediction model of suicidal ideation. We used a Decision Tree Analysis using SPSS 23.0 program to explore predictors of suicide thoughts for 12,015 Korean adults aged 19–98 years. As a result, the most powerful predictor of suicidal ideation was the level of depression. Of people who suspected depression (CESD-11>16), 32.6% experienced suicidal ideation, which is 12 times higher than that of total subjects. The group with the highest rate of suicidal ideation was people who experienced financial difficulties in depression-suspected group and the rate of suicidal thoughts in this group was 56.7%, which was the highest rate. However, in the non-depressive group, the satisfaction of family relationship was the strongest predictor of suicidal ideation. In the non-depressive group, the rate of suicidal thoughts of people with high level of family relationship satisfaction and high level of health satisfaction was 0.6%, which was the lowest rate. The contribution of this study was that it provided the combination of variables to predict the risk groups of adult suicide. This study suggests that researchers and clinicians should consider comprehensively depressive symptoms, family relationships, economic difficulties, and health status to prevent the suicide of adults.

## Introduction

Suicide is one of the leading causes of death worldwide. In particular, the suicide rate of Korean adults is around 30 per 100,000 people [1]. A possible explanation for the high suicide rate of Korean society may be due to the lack of a systemic clinical approach to predict suicidality.

One major predictor of adult suicidal ideation is psychiatric problems such as depression and alcohol abuse [2–5]. In particular, depressive symptom is a strong predictor of suicidal idea and suicide attempt [6,7].

Other major predictors of suicidal ideation are life stressors [2]. Adverse childhood experiences such as parental death and divorce, academic suspension, and physical/sexual abuse may affect adulthood suicidal ideation [8]. Especially, childhood trauma such as physical and sexual abuse may lead to depressive symptoms, which, in turn, may affect suicidal thoughts and suicide attempts [9].

In particular, interpersonal problems are likely to cause suicidal ideation and suicide attempts. According to the interpersonal theory of suicide [10], social connectedness is one of the basic needs of human. When it is deficient, people may experience suicidal thoughts. Especially, family cohesion and family conflict have been identified as important predictors of suicide [5,11]. In addition, the single household family might induce suicidal ideation because they may lack social connectedness [12].

Economic stressors also may lead to suicidal thoughts [13,14]. Economic stress may induce negative emotions such as depressed mood, disappointment, which may in turn induce suicidal ideation and suicide attempts [15].

Physical health is may be related to suicide, but previous studies have not fully considered it. The presence of physical disorder and physical disability may also affect mental health, and might lead to suicidal ideation [16,17]. Especially, physical disease such as cancer, heart disease, and liver disease and physical disability are closely related to depression, and consequent to suicidal ideation and suicide attempts [18].

Work environment and housing may also be related to life satisfaction. In particular, the housing in Korean society (e.g., types of housing) is closely related to life satisfaction. In previous studies, work environment such as types of employment (permanent position or temporary position), unemployment, and job stress were found to be associated with life satisfaction, and life satisfaction was negatively associated with suicidal ideation [19,20].

The major stressors mentioned above are related to life satisfaction, which is an important predictor of suicide found in previous studies [21,22]. Previous studies have examined the impact of overall life satisfaction on suicide, but efforts to separately analyze the effects of life satisfaction on suicidal ideation in the main areas of life such as health, occupation, and family relationships need to explore the optimal combination predicting suicidal ideation.

Sex [23], age [24], academic background [17], and marital status [16] were found to be prominent predictors of suicidal ideation in previous studies. In particular, income is consistently identified as a key predictor of suicidal ideation and suicide attempts in a number of studies [25]. A study by Hung, Kwok, Yip, Gunnell and Chen (2015) of Taiwanese adults, a community-based cohort study, found that lower income was positively related to risk of suicide.

In sum, previous studies have identified the independent effects of predictors of suicidal ideation, and most studies did not consider major predictors together. Based on the Cry of Pain model [26], experiencing multiple stress than a single stress may greatly increase suicidal ideation. Thus, in order to more accurately predict suicide ideation, socio-demographic variables and key predictors should be considered together [27]. In particular, developing a predictive model by a combination of risk factors may provide more information in preventing suicide than verifying the independent effects of risk factors.

The aim of this study is to investigate the prediction model of adult suicidal ideation using Decision Tree Analysis. Decision Tree analysis generally aims to find optimal combinations that predict target variables based on large quantities of data, rather than other traditional method that set and verify hypotheses.

## Materials and methods

### Participants

This study analyzed data from the Korean Welfare Panel Survey (2016) by the Korea Institute for Health and Social Affairs. The Korea Welfare Panel Survey was conducted in 209 cities and districts in 17 provinces for 90 days from March 2 to June 9, 2016. The survey was conducted by face-to-face interview of 50 trained interviewers visiting the client's household. In the first step of the sampling, 517 survey sites were sampled from the 2005 census and the household income and the economic activity of household members were identified. In the second stage, a total of 7,000 households were sampled from the first stage data, with 3,500 general households and low-income households respectively. In the final stage, final panel households were selected using a stratified extraction method. In this study, data from 12,015 adults aged 19 or older were used for analysis.

### Measures

#### Socio-demographic variables

Gender, age, educational background (uneducated, elementary school, middle school, high school, college, university, master, and doctoral graduate), marital status (married, bereavement, divorce, separation, and unmarried), and religion (yes or no), and monthly income (Table 1).

**Table 1:** Socio-demographic characteristics.

Variable	Variable	Frequency	Percent
Gender	Male	5240	42.8
	Female	6875	57.2
Age group	19–39	2323	19.3
	40–64	4819	40.1
	>64	4873	40.6
Education	Uneducated	1050	8.7
	Elementary school	2525	21.0
	Middle school	1474	12.3
	High school	3287	27.4
	College	1208	10.1
	University	2184	18.2
	Master	246	2.0
	Doctor	41	0.3
Marital State	Married	7579	63.1
	Bereavement	1954	16.3
	Divorced	664	5.5
	Separation	78	0.6
	Unmarried	1739	14.5

N = 12,015

#### Childhood trauma

Parental death (yes or no), suspension of studies (yes or no), and divorce of parents (yes or no).

#### Health information

The presence of disorder (physical disorders: physical disability, brain lesions, hearing disorders, speech disorders, kidney disorders, cardiac disorders, respiratory disorders, liver disorders, facial disorders, epilepsy disorders; mental disorder: mental retardation, psychotic disorders, and developmental disorders; none of the above), and disability grade (from 1 to 6).

#### Work information

Working ability (capacity for work, capacity for simple work, incapacity for work), employment status (employed, self-employed/employer, and unemployed).

#### Housing information

Living alone (yes or no), housing types (owner occupied housing, key money deposit, monthly rent).

#### Economic information

Low income households (yes or no), household debt.

#### Financial difficulties

Financial difficulties were measured through eight items with discrete scale (yes or no). The specific items are as follows: the experience of moving home because the rent cannot be paid for more than 2 months, experience of not paying bills in due time, no electricity/telephone/water due to not paying taxes, experience of not paying more than one month for public education of child, the experience of not going to the hospital due to lack of money, the experience of becoming a credit delinquent among family members, and experience of suspension of insurance benefits due to non-payment of health insurance. The Cronbach Alpha for the data used in this study was .70.

#### Life satisfaction

Life satisfaction was measured by health, family income, housing environment, occupation, family relationship, and social relationship. Each area was measured as a single item on a 5-point scale (very unsatisfactory = 1, very satisfied = 5). Example of specific item is as follows. Are you satisfied with your household income?

#### Depression

CESD-11(Center for Epidemiologic Studies-Depression Scale 11) was used to measure depressive level. CESD-11 is an abbreviated version of the CESD developed by Radloff (1977). It consists of 11 items with 4-point scale (extremely rare, occasionally, often, most). Higher score is related to higher level of depression. People with a score of 16 out of a total of 33 points are likely to be diagnosed with depression, and we named this group as a depression-suspected group [28]. The Cronbach Alpha for the data used in this study was .89.

#### Alcohol dependence

This study used the World Health Organization's Alcohol Use Disorder Identification Test (AUDIT) to determine alcohol dependence. AUDIT consists of 10 questions and measures three areas (harmful drinking, alcohol dependence, and dangerous drinking). A score of 20 or higher is a condition requiring treatment with alcohol dependence [29]. The Cronbach Alpha for the data used in this study was .82.

#### Suicidal ideation

The participants were asked the following questions. ‘Have you ever experienced suicidal thoughts in the past year? Participants answered yes or no.

### Analysis

We used SPSS 23.0 program to perform Decision Tree Analysis. This Analysis is an effective method for classification and prediction, and especially predicts effectively the target variable coded with nominal scale by making the rules that classifies the target object into several subgroups of a tree structure [30]. In addition, it can effectively analyze interactions between continuous variables and discrete variables [31]. We used a Chi-squared automatic interaction detection (CHAID; Magidson, and SPSS Inc 1993) method as the separation (growing) method. CHAID is suitable for predicting dichotomic target variables and to conduct multiple split using chi-square verification. Multiple split is the separation of two or more child nodes from the parent node. In the stopping rules, the depth of the tree was set at four levels, and the minimum number of cases of the parent node and the child node were set to 100 and 50 respectively. We used the Gains and Risk charts to determine the fit of the model. Gains chart is the percentage of the target category (experience of suicidal thought) in each node. The model of the decision tree is chancy, even with small variations in the training dataset. One effective way to solve this problem is to use random forests. Thus, we used the k-fold cross validation method for random forests and established the number of sample folds as 10 groups.

## Results

**Fig 1:**
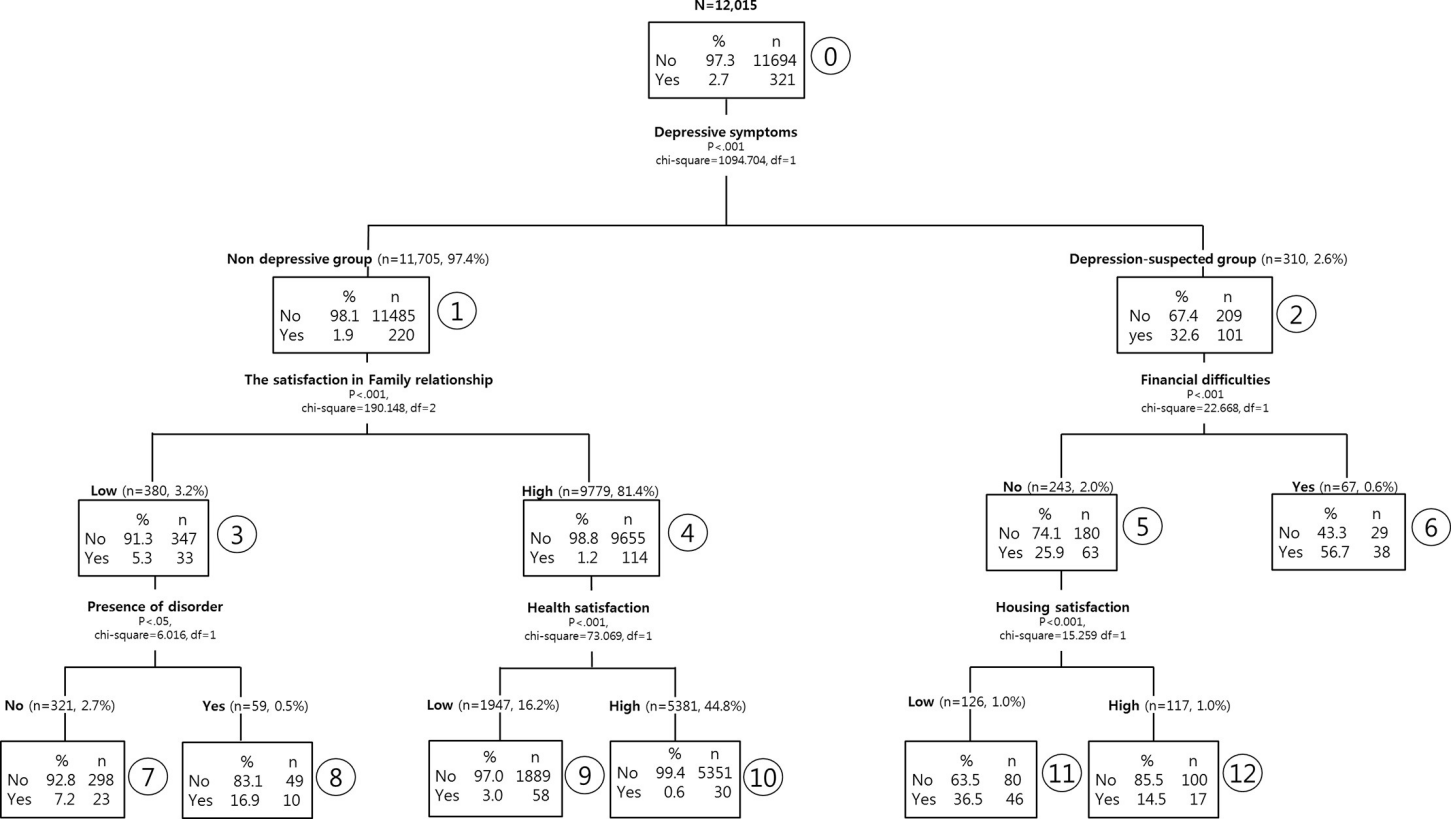
Decision tree of suicidal thoughts in adults group.

Decision tree of suicidal thoughts is shown in Fig 1. Of the total samples, 2.7% (321 people) experienced suicidal ideation, while 97.3% (11,694 people) did not experience suicidal thoughts. Decision tree analysis showed that the most powerful predictor of adult suicidal thoughts was depressive symptoms (Chi-square = 1094.794, *p*<0.001). Of the 321 people suspected of depression (CESD-11>16), 101 people (32.6%) reported suicidal ideation. However, 220 people (1.9%) of the non-depressed group (11,485 people) experienced suicidal thoughts.

The strongest predictor of suicidal thoughts in the depression-suspected group was financial difficulties (Chi-square = 22.668, *p*<0.001). The rate of suicidal ideation of 67 people who experienced economic difficulties in depression-suspected group was 56.7% (38 people) which was the highest rate, while the rate of suicidal thoughts of 243 people who did not experience financial difficulties in depression-suspected group was 25.9% (63 people). In a group who suspected of depression, but had no experience of financial difficulties, housing satisfaction was the most important predictor of suicidal ideation (Chi-square = 15.259, *p <* .01). Of 117 people who were suspected depression and had no experience of financial difficulties, and housing satisfaction was high, the rate of suicidal thoughts was 14.5% (17 people). On the other hand, in the group of 126 people who were suspected depression, and had no experience of financial difficulties, and had a low satisfaction of housing, the rate of suicidal ideation was 36.5% (46 people).

In the non-depressed group, the most powerful predictor of suicidal ideation was satisfaction with family relationship (Chi-square = 190.148, *p*<0.001). In the non-depressed group, the rate of suicidal ideation of 9,779 people with high satisfaction with family relationship was 1.2% (114 people). However, the rate of suicidal thoughts of 380 people with low satisfaction with family relationship was 8.7% (33 people) and the rate of suicidal thoughts of 1,546 people with middle satisfaction with family relationship was 4.7% (73 people). In the group of people who are not depressed and have a high satisfaction with family relationship, health satisfaction was the most powerful predictor of suicidal ideation (Chi-square = 73.069, *p* < .001). Especially, in the group of 5,381 people who are not depressed and have a high satisfaction with family relationship and have a high satisfaction with health, the rate of suicidal ideation decreased to 0.6% (30 people), which was the lowest rate. In the group of people who are not depressed and had a low satisfaction with family relationship, the presence of disorder was the most powerful predictor of suicidal ideation (Chi-square = 6.016, *p*<0.5). In the group of 59 non-depressed participants with a low satisfaction with family relationship and the presence of disorder, the rate of suicidal thoughts was 16.9% (10 people).

The gain chart in Table 2 demonstrates the predictive power of each model for the target category. Node-by-nodes are arranged in descending order of gain score. In this study, the node with the highest gain score is node 7, which is the group with financial difficulties and who are suspected depression. Of the total 12,015 people, 67 subjects belong to node 7, which is 0.6% of total subjects. The number of cases (gain: n) corresponding to the target category among the 67 subjects belonging to the sixth node is 38, which is 56.7% (Resp: %) of the total cases of the node. The index of node 7 is 2122.9%. There is no clear standard for gain Index, but if the Index of a specific node is more than 200%, the gain score is very high.

**Table 2:** Gain index of predicting suicidal ideation.

Node	Node		Gain		Response (%)	Index (%)
	N	percent	N	percent		
6	67	0.6	38	11.8	56.7	2122.9
11	126	1.0	46	14.3	36.5	1366.5
8	59	0.5	10	3.1	16.9	634.4
12	117	1.0	17	5.3	14.5	543.9
7	321	2.7	23	7.2	7.2	268.2
9	1947	16.2	58	18.1	3.0	111.5
10	5381	44.8	30	9.3	0.6	20.9

Note: Node, each node number; Node(n), sample size of each node; Node(%), rate of target category of total sample; Gain(n), sample size of target category of each node; Gain(%), rate of target category of each node of total target category; Resp(%), rate of sample size of target category of sample size of each node; Index(%), Resp(%) versus rate of target category of sample size.

We conducted the k-fold cross validation test for the cross validation in the final model. The results indicated that the risk estimates (.026) and standard errors (.001) of the model applying the cross-validation test did not differ from the results of the model without the cross-validation test (Table 3). This can be interpreted as the stability of the model being guaranteed.

**Table 3:** The risk estimation.

	Cross validation	Resubstitution
Estimate	0.026	0.026
Standard error	0.001	0.001

## Discussion

The purpose of this study was to explore the optimal combination predicting adult suicidal ideation using Decision Tree Analysis.

Depressed symptom was the strongest predictor of adult suicidal ideation. Importantly, 56.7 percent of people who experienced financial difficulties in the depression-suspected group experienced suicidal thoughts. In previous studies, depression and economic factors such as economic stress, financial difficulties were important predictors of adult suicide [15,32,33]. This study showed that the combination of depression and financial difficulties dramatically increased the rate of suicidal ideation [34].

In the non-depressed group, satisfaction with family relationship was the strongest predictor of suicidal ideation. According to Interpersonal Psychological Theory of Suicide [35], thwarted belongingness and burdensomeness due to family conflicts may cause suicidal ideation. In addition, this result implies that interpersonal stress may be more related to suicidal ideation than non-social stressors (e.g., housing, financial difficulties) in the non-depressed group [36].

In the model, the combination of the highest rate of suicidal thoughts was depressive symptoms and economic difficulties. On the other hand, in the non-depressed group, the highest rate of suicidal ideation was detected in the combination of low satisfaction with family relationship and the presence of disorder. These results suggest that experiencing of multiple stressors may increase the likelihood of suicidal ideation. According to the Cry of Pain model [26], multiple stressors in various areas of life may greatly increase suicidal ideation [13,15,37].

In the depression-suspected group, the rate of suicidal ideation of people with low satisfaction with housing but have no financial difficulties was 36.5%, which was the second highest rate. This group is estimated to be the House-Poor. That is a situation that describes people who spend a large proportion of their total income on home ownership (e.g., mortgage payments, property taxes). These results suggest that low satisfactory with housing in the depression-suspected may induce strong stress, which may in turn lead to suicidal ideation [15].

In the non-depressed group with high satisfaction of family relationship, health satisfaction was important predictors of suicidal ideation. In previous studies, health-related factors (e.g., perceived health status, physiological disease) were identified as major stressors causing suicidal ideation [38]. Low satisfaction with health may trigger depressed mood and may consequently cause suicidal thoughts. In particular, in the non-depressed group, the lowest rate of suicidal ideation was found in the combination of high satisfaction with family relationships and high satisfaction with health.

The results of this study suggest that it is necessary to continually monitor the depressed group experiencing financial problems to decrease suicidal idea and in order to increase the prediction power of suicide, it is important to try to find a combination of risk factors of suicidal ideation and suicide attempts.

The contribution of this study is to verify the combination of factors predicting adult suicide using a Decision Tree Analysis for nationwide sample. The limitations of this study and suggestions in future studies are as follows. This study did not consider suicidal attempt that is more directly related to suicide behavior or completed suicide. In future studies, researchers need to develop a model that predicts suicide by including suicide plan and suicide attempt.

There are numerous studies that evaluated risk of suicide and the suicide rate increased when a person suffers from multiple stresses, such as trauma, homelessness, history of violence, psychiatric illness [39, 40]. There is a clear addictive effect when an individual suffers from multiple factors. In addition, this study also did not include specific information related to depression such as period of depression (acute or chronic) and recurrence [12] and protective factors which are also extremely important in determining suicide risk such as social support. More factors need to be added to the decision tree in future studies.

In future studies, the development of a clinical tool to incorporate risk factors and protective factors to determine risk of suicide when evaluating the need for involuntary commitment is necessary. Furthermore, past studies have focused on individual factors such as depression. However, the problems of the social structure such as the national economic crisis and the gap between the rich and poor may directly or indirectly affect suicide.

## Conclusions

The results of this study suggest that various factors such as depression, economic difficulty, family relationship, and perceived health status should be considered in predicting adult suicide. Especially, this study implies that financial difficulties in depression-suspected group and family relationship in non-depressive group should be considered for adult suicide prevention.
